# A Root-Preferential DFR-Like Gene Encoding Dihydrokaempferol Reductase Involved in Anthocyanin Biosynthesis of Purple-Fleshed Sweet Potato

**DOI:** 10.3389/fpls.2017.00279

**Published:** 2017-02-28

**Authors:** Xiaoqiang Liu, Min Xiang, Yufang Fan, Chunxian Yang, Lingjiang Zeng, Qitang Zhang, Min Chen, Zhihua Liao

**Affiliations:** ^1^Key Laboratory of Eco-environments in Three Gorges Reservoir Region – Ministry of Education, Chongqing Engineering Research Centre for Sweet Potato, School of Life Sciences, Southwest UniversityChongqing, China; ^2^College of Pharmaceutical Sciences, Southwest UniversityChongqing, China

**Keywords:** anthocyanin biosynthesis, dihydrokaempferol reductase, functional identification, *Ipomoea batatas*, transgenic

## Abstract

Purple-fleshed sweet potato is good for health due to rich anthocyanins in tubers. Although the anthocyanin biosynthetic pathway is well understood in up-ground organs of plants, the knowledge on anthocyanin biosynthesis in underground tubers is limited. In the present study, we isolated and functionally characterized a root-preferential gene encoding dihydrokaempferol reductase (*IbDHKR*) from purple-fleshed sweet potato. IbDHKR showed highly similarity with the reported dihydroflavonol reductases in other plant species at the sequence levels and the NADPH-binding motif and the substrate-binding domain were also found in IbDHKR. The tissue profile showed that *IbDHKR* was expressed in all the tested organs, but with much higher level in tuber roots. The expression level of *IbDHKR* was consistent with the anthocyanin content in sweet potato organs, suggesting that tuber roots were the main organs to synthesize anthocyanins. The recombinant 44 kD IbDHKR was purified and fed by three different dihydroflavonol substrates including dihydrokaempferol (DHK), dihydroquerctin, and dihydromyrecetin. The substrate feeding assay indicated that only DHK could be accepted as substrate by IbDHKR, which was reduced to leucopelargonidin confirmed by LC-MS. Finally, IbDHKR was overexpressed in transgenic tobacco. The IbDHKR-overexpression tobacco corolla was more highly pigmented and contained higher level of anthocyanins than the wild-type tobacco corolla. In summary, *IbDHKR* was a root-preferential gene involved in anthocyanin biosynthesis and its encoding protein, specifically catalyzing DHK reduction to yield leucopelargonidin, was a candidate gene for engineering anthocyanin biosynthetic pathway.

## Introduction

Sweet potato (*Ipomoea batatas* Lam.) is one of the most important crops in the world, ranking fifth among the most important food crops, after rice, wheat, maize and cassava (Fu et al., 2014). As one of the most healthful food recommended by Food and Agriculture Organization, sweet potato contains starches, dietary fiber and healthful metabolites including β-carotene and anthocyanins. Among sweet potato cultivars, the purple-fleshed sweet potato is particularly nutritionally valuable due to rich anthocyanins in storage tuber roots (Hu et al., 2016). Purple-fleshed sweet potato has higher economic value for the farmers especially in developing countries, because purple sweet potato not only produces starch but also yields high-value anthocyanins widely used as natural food additives and flavors. The sweet potato breeders have been paid much attention to develop purple-fleshed sweet potato varieties, but genetic breeding is time-consuming. Alternatively, metabolic engineering might be a promising method to develop new varieties of purple-fleshed sweet potato. Anthocyanins are a large group of water-soluble flavonoids widely distributed in higher plants (Sun et al., 2016). The regulation of anthocyanin biosynthesis is relatively clear in the above-ground organs such as seeds, flowers and leaves, but it is not clear in the underground organs such as sweet potato storage tuber roots that grow in dark conditions (Mano et al., 2007). The purple-fleshed sweet potato is an ideal plant species for studying anthocyanin biosynthesis in underground organs.

**FIGURE 1:**
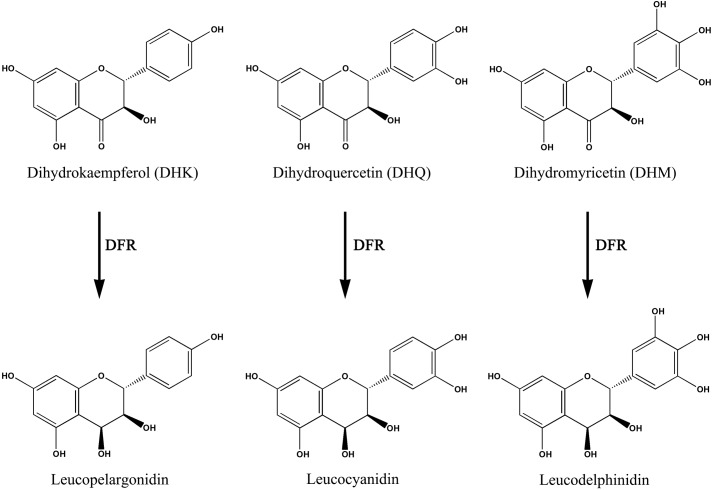
**Being a key enzyme of the anthocyanins biosynthetic pathway, the dihydroflavonol reductase (DFR) catalyzes the NADPH-dependent reduction of dihydroflavonols to corresponding products.** DFR, dihydroflavonol 4-reductase.

Dihydroflavonol reductase (DFR) is a multifunctional enzyme involved in anthocyanin biosynthesis, which selectively or unselectively reduces dihydroflavonols such as dihydrokaemp ferol (DHK), dihydroquerctin (DHQ) and dihydromyrecetin (DHM) to corresponding products (**Figure 1**). In *Gerbera hybrida*, the DFR enzyme (GhDFR) catalyzes the reduction of all the three substrates, but petunia DFR only accepts DHQ and DHM as substrates (Johnson et al., 1999). DFR is also the key enzyme involved in anthocyanin biosynthesis, which is often used to engineering the biosynthetic pathway of anthocyanins. Overexpression of DHK-preferring GhDFR in white petunia made the flower color become red due to leucopelargonidin-related anthocyanin accumulation (Meyer et al., 1987). Wang et al. (2013) reported a DFR gene (IbDFR) from purple-fleshed sweet potato that was expressed in anthocyanin-synthesizing organs including leaves, tuber roots and other organs. Suppressing IbDFR led to the decrease of anthocyanin accumulation and reduced the tolerance on abiotic stress of sweet potato (Wang et al., 2013). In our study, a root-preferential DFR-like gene was functionally identified to specifically catalyze DHK reduction and overexpression of this gene promoted anthocyanin accumulation in transgenic tobacco flowers.

## Materials and Methods

### Plant Materials

The purple-fleshed sweet potato cv. YUZI263 was used to clone the gene and analyze gene expression and anthocyanin contents. Sweet potato YUZI263 were planted in the field and 5-month-old plants were harvested and frozen in liquid nitrogen for future using. Total RNAs and anthocyanins were extracted from storage roots (Φ 0.5 cm, Φ 3.0 cm), veins, petioles, leaves and vein tips. *Nicotiana tabacum* was used as plant materials for genetic transformation.

### Gene Cloning and Expression Analysis

The total RNAs of tubers were used as initial material for gene cloning. The full-length cDNA of *IbDHKR* was obtained using rapid amplification of cDNA ends (RACE) technology. A pair of primers, *fibdhkr-core* (5′-CTGCTGGCTTTATCGGCTCC-3′) and *ribdhkr-core* (5′-GGCACTGTTGTGGCCTCTTG-3′), were used to amplified the core fragment of *IbDHKR*. The 3′ end of *IbDHKR* was amplified using a gene-specific primer *ibdhkr3* (5′- GGAAGAAGGGGTTACTTCCCTA-3′) and NUP (5′-AAGCAGTGGTATCAACGCAGAGT-3′). The 5′ end of *IbDHKR* was amplified using a gene-specific primer *ibdhkr5* (5′-GGATCCTTGGAATCGAAATCCATA-3′) combined with NUP. The coding sequence of the full-length *IbDHKR* was amplified using a pair of primers, *fibdhkr* (5′- ATGGTGGACGGTAATCATCCAA-3′) and *ribdhkr* (5′- TCAAGCTTTTAAGGGCACTACC -3′). Homology and sequence identity were confirmed using the BLAST program.

#### The Tissue Profile of IbDHKR was Analyzed by Real-Time Quantitative PCR (qPCR)

Real-time quantitative PCR (qPCR) was performed using two step qPCR kit, the PrimeScript^TM^ RT Reagent Kit and SYBR Premix ExTaq^TM^ (TaKaRa, Japan). Pure mRNA was prepared from the frozen plant organisms using the RNA simple Total RNA Kit (TIANGEN, Beijing). Reverse transcription of mRNA (500 ng) was achieved by following the manufacturer’s protocol. The 18S gene, which has been reported to be constitutively expressed, was used as an endogenous reference. Primers were: QF-IbDHKR (5′-TGGTGGACGGTAATCATC-3′), QR-IbDHKR (5′-CGCTTTCGGTAGTTCAAG-3′), QF-IbF3H (5′-TGAAGGCGTGTGAAGATTGG-3′), QR-IbF3H (5′-CGGCAAGGCGAAGAAGTC-3′) and the QF-18S (5′-CAGATACCGTCCTAGTCTCAAC-3′), QR-18S (5′-CAGCCTTGCGACCATACTC-3′). The relative quantification of *IbDHKR* expression between organisms was calculated using the comparative threshold (CT) method (Heid et al., 1996).

### Detection of Anthocyanin Contents

Anthocyanins were extracted according to the reported proce dures with minor modification. The solution containing anthocyanins was measured by UV Spectrophotometer U-1100 (HITACHI, Japan), the absorbance at 530 nm was measured three times and the average values were represented for the relative contents in different tissues.

### Purification of Recombinant Protein and Substrate-feeding Assay

Competent *Escherichia coli* cells of the strain BL21 (DE3) were transformed with the pET28a(+)-*IbDHKR* constructs. The *E. coli* cells were subcultured to a density of OD_600_ = 0.5 in Luria–Bertani medium containing kanamycin (100 mg/l). IPTG was added to a final concentration of 0.5 mM for the induction of protein expression, and the bacteria were harvested following an additional 16 h of growth at 16°C and stored frozen at –70°C. The recombinant IbDHKR proteins were partially purified from crude bacterial extracts and monitored by SDS-PAGE and detection with the Anti-His antibody and visualized using WesternBreeze^TM^ Chemiluminescent kit (Invitrogen, Carlsbad, CA, USA), the Western blot analysis was preferred according to the kit manufacturer’s protocol. The 6× His tagged recombinant proteins were purified using Ni-NTA columns under native conditions. Substrate-feeding assay was conducted according to Xie et al. (2004) with some modifications. The initial standard assay conditions included incubation at 30°C for 30 min in 100 μl total volume containing 48 μl of 100 mM Tris HCl (pH7.0), 40 μl of 0.45 μg/μl IbDHKR purified protein, 10 μl of 10 mM NADPH, and 2 μl of 10 μg/μl substrate (DHK). Enzyme reactions were stopped by adding 200 μl of ethyl acetate and vortexing. Samples were centrifuged for 2 min at 10,000*g* and 150 μl of ethyl acetate extract was removed to a new tube and evaporated with a stream of nitrogen gas. Residues were dissolved in 30 μl of methanol and used directly for LC/MS analysis. An Agilent 6320 ion trap LC/MS system was used under default Smart Parameter settings. The analyzer and ion optics were adjusted to achieve optimal resolution using the ESI Tuning Mix. The mass spectrometer was scanned in the m/z range of 50–2200 at 8100 mass units/s with an expected peak width of ≤0.35 mass units. For automated MS/MS, the trap isolation width was four atomic mass units. The associated Agilent 1200 LC was fitted with a Zorbax 80A EXTEND-C18 column (150 mm × 2.1 mm, 5 μ particle size) maintained at 35°C. The binary solvent system consisted of 90:10 (v/v) water/acetonitrile containing 0.1% formic acid and 0.1% ammonium formate (solvent A) and 10:90 (v/v) water/acetonitrile containing 0.1% formic acid and 0.1% ammonium formate (solvent B). The separation gradient was 100% A to 100%B in 4.5 ml over 30 min.

### Establishment of Transgenic Tobacco

The coding regions from *IbDHKR* was subcloned into the *Bgl* II and *Bst*E II site of the binary vector pCAMBIA1304^+^ (Kai et al., 2009), replacing the *mGFP5* and *gusA* genes. The pCAMBIA1304^+^-*IbDHKR* derivative and un-modified pCAMBIA1304^+^ were introduced into *Agrobacterium tumefaciens* EHA105 bacteria and used to transform tobacco leaf disks. Transformation and regeneration of tobacco leaf disks used the common methods. Agrobacterium was cleared from the explants using 250 mg/ml carbenicillin. The disks were cultured on the MS containing 0.1 mg/L NAA, 1.0 mg/L 6-BA, 250 mg/L carbenicillin (Cb), and 20 mg/L hygromycin (Hyg) at 25°C underlight for one month, then subcultured on MS with 250mg/L Cb and 20mg/L Hyg for 4 to 5 weeks to regenerate shoots. Subsequently, the regenerated shoots were excised from the original explants and cultured on MS (IBA 0.1 mg/L + Hyg 20 mg/L + Cb 250 mg/L) to grow to plants. Mature plants were transferred to pots and maintained under standard greenhouse conditions. The transgenic plants were checked by PCR method using the primers: F-gp (5′- ACTATCCTTCGCAAGACCCT-3′) and R-gp (5′- ATCACTCCAGCAGCTCTCATC-3′).

## Results

### Cloning and Sequence Analysis of *IbDHKR*

**FIGURE 2:**
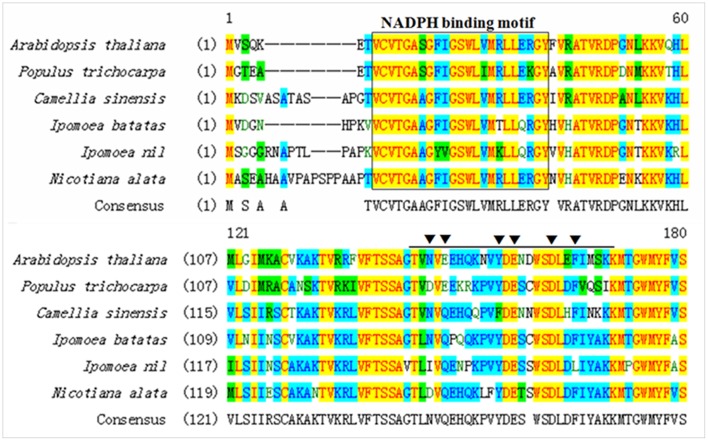
**Multiple alignments of the NADPH-binding motif (in black box) and the substrate-binding domain of DFR proteins (in black arrow)**.

**FIGURE 3:**
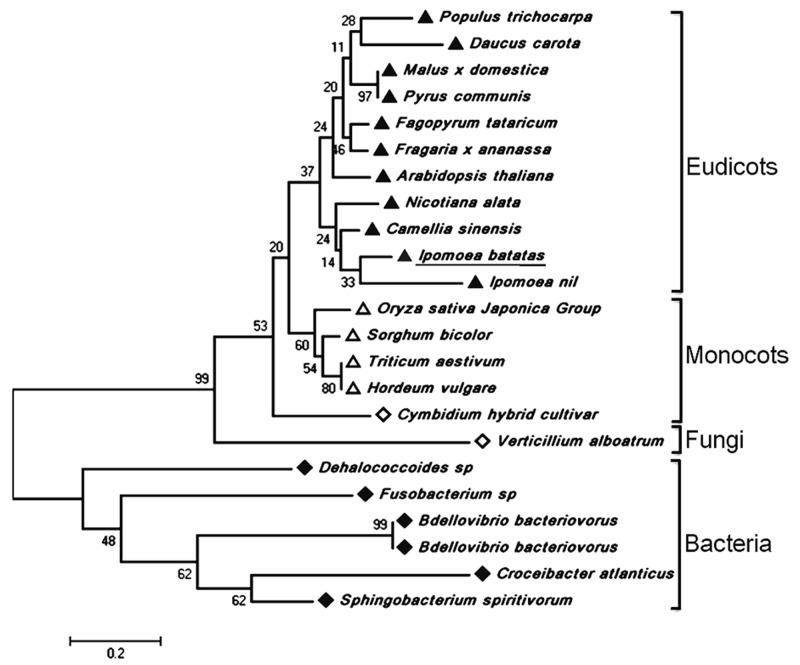
**The phylogenetic analysis of DFR proteins.** The numbers represented the bootstrap values.

The full-length cDNA of *IbDHKR* (GenBank accession No. HQ441167) was 1392 bp in length with a 1185-bp coding sequence encoding a 394-amino-acid polypeptide with the calculated molecular weight of 44 kD. The BLAST searching results indicated that IbDHKR showed higher similarity with the DFRs from *Ipomoea nil* (94% similarity), *Nicotiana alata* (77% similarity), and *Arabidopsis thaliana* (58% similarity). The NADPH-binding motif (V10-Y32) (Shimada et al., 2005), as well as the substrate-binding domain (T133-K158) (Beld et al., 1989; Johnson et al., 2001), was found in IbDHKR according to the multiple alignments (**Figure 2**). The homology-based modeling showed that the structure of IbDHKR was very similar with the grape DFR protein, the NADPH/substrate-binding motif exposed on the surface (Petit et al., 2007). The phylogenetic analysis indicated that the plant DFRs were divided into eudicot type and monocot type because the DFRs from eudicots plants formed a clade separate from the clade composed of monocot DFRs (**Figure 3**). The bioinformatic analysis suggested that IbDHKR belonged to the DFR family.

### Tissue Profile of Anthocyanin-Biosynthetic Genes and Anthocyanin Accumulation

**FIGURE 4:**
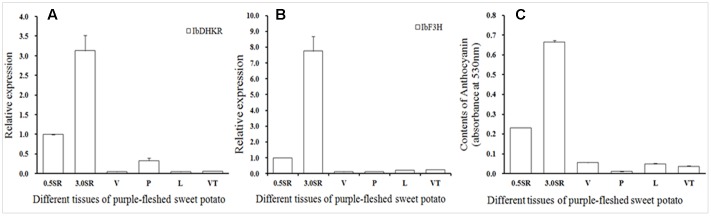
**The tissue profiles of IbDHKR (isolated and functionally characterized a root-preferential gene encoding dihydrokaempferol reductase)** **(A)** and IbF3H **(B)** and anthocyanin accumulation **(C)**. SR: storage root; V, vein; P, petiole; L, leaf; VT, vein tip.

Purple-fleshed sweet potato YUZI263 is the first cultivar authorized by China Ministry of Agriculture, which has high-yield starch and contains rich anthocyanins in tubers (Zeng et al., 2013). The tissue profiles of two anthocyanin-biosynthetic genes including *flavanone 3-hydroxylase* (*IbF3H*) (**Figure 4A**) and *IbDHKR* were analyzed in different organs by qPCR (**Figure 4B**). *IbDHKR* expression level was much higher in storage roots (Φ 3.0 cm or 0.5 cm) than in up-ground organs including veins, petioles, leaves and vein tips. For example, the expression level of *IbDHKR* was about 9.4-fold higher in storage roots (Φ 3.0 cm) than in petioles. Notable, bigger storage roots (Φ 3.0 cm) had significantly higher expression level of *IbDHKR* (3.1-folds) than slimmer storage roots (Φ 0.5 cm), suggesting that *IbDHKR* expression was associated with YUZI263 storage root development. For sweet potato *IbF3H*, it was also highly expressed in storage roots, just like the tissue profiles of *IbDHKR*. The previous research reported that *anthocyanidin synthase* (*IbANS*) of YUZI263 was expressed preferentially in storage roots in which the anthocyanin content was much higher than other organs (Liu et al., 2010). The root-preferential tissue profiles of anthocyanin-biosynthetic genes suggested that storage roots were the main organs to synthesize anthocyanins. The coordinate expression of the anthocyanin-biosynthetic genes facilitated anthocyanin biosynthesis in storage roots. Analysis of the anthocyanin contents in different organs indicated that storage roots also contained much higher level of anthocyanins than any other organ (**Figure 4C**). The anthocyanin content in storage roots (Φ 3.0 cm) was about 10–58 folds of that in up-ground organs. The storage roots with 3 cm diameter had higher level of anthocyanins than storage roots with 0.5 cm diameter, suggesting that anthocyanin accumulation was positively linked with development of storage roots. These results on analyzing gene expression and anthocyanin accumulation showed that the anthocyanin accumulation was strongly in accordance with the expression levels of anthocyanin-biosynthetic genes in purple-fleshed sweet potato YUZI263.

### Purification of Recombinant IbDHKR and Substrate-Feeding Assay

**FIGURE 5:**
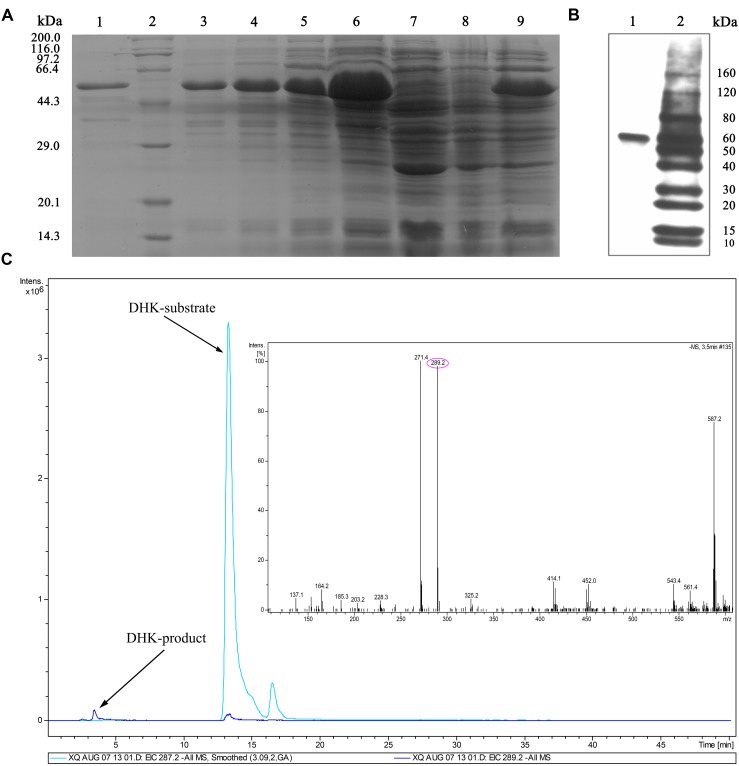
**Detection of recombinant IbDHKR using western blot and substrate-feeding assay.** **(A)** Purification of recombinant IbDHKR from engineered *Escherichia coli*. (1) The purified recombinant IbDHKR; (2) Protein marker; (3–6) the IPTG-induced cells from 1 to 4 h; (7) *E. coli* harboring pET28a(+); (8) *E. coli* harboring pET28a(+)-*IbDHKR* without IPTG induction; (9) the supernatant of *E. coli* harboring pET28a(+)-*IbDHKR* with IPTG induction. **(B)** Detection of recombinant IbDHKR using western blot. (1) The result of western blot assay on IbDHKR; (2) Protein marker. **(C)** Confirmation of the product of IbDHKR using LC-MS.

To unveil the stereospecific reduction, the recombinant protein of His-tagged IbDHKR was purified from engineered *E. coli*. When engineered *E. coli* was induced by IPTG, a specific 44 kD protein band was found, but this band was not found in engineered E. coli (pET28a(+)-*IbDHKR*) without IPTG treatment and in *E. coli* with the blank vector pET28a(+). With the extension of IPTG induction time, this protein band became thicker and thicker. In 4-hour induction of *E. coli* by IPTG, engineered *E. coli* produced the specific protein of which quantity was enough for analysis (**Figure 5A**). After harvesting ultrasonically broken *E. coli* using centrifuge, this protein was also found in supernatant of the broken debris of engineering *E. coli* (**Figure 5A**), facilitating protein purification. Then, the recombinant His-tagged IbDHKR was purified using Ni^2+^ column, of which molecular weight was about 44 kD (**Figure 5B**), consistent with the calculated molecular weight. The molecular weight of IbDHKR was similar with the reported DFRs from *Camellia sinensis* (L.) O. Kuntze (Singh et al., 2009), *Medicago truncatula* (Xie et al., 2004) and *Vitis vinifera* (L.) (Petit et al., 2007). Then, the recombinant His-tagged IbDHKR was detected using antibody against His-tags by western blot (**Figure 5B**). Finally, three different types of dihydroflavonol substrates including DHK, DHQ and DHM were, respectively, fed to the recombinant IbDHKR. The results indicated that IbDHKR catalyzed the reduction of DHK alone to produce leucopelargonidin that was confirmed by LC-MS (**Figure 5C**). When DHQ or DHM was fed to recombinant IbDHKR, no products were detected. These results suggested that this recombinant protein was a dihydroflavonols reductase specifically reducing DHK (dihydrokaempferol reductase) to produce leucopelargonidin.

### Overexpression of *IbDHKR* in Tobacco

**FIGURE 6:**
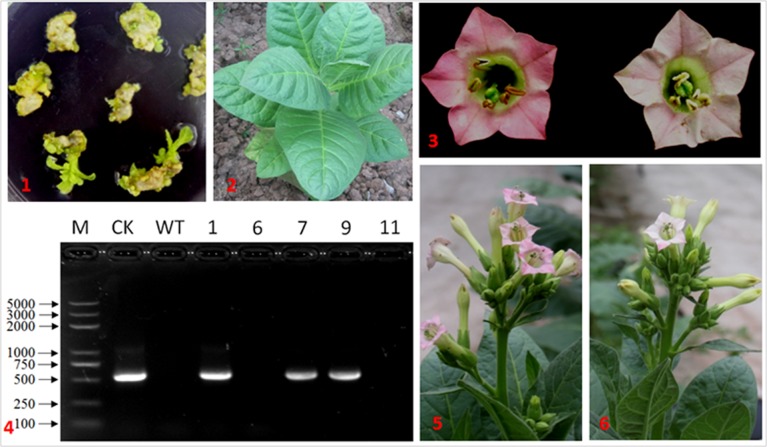
**IbDHKR-overexpressing tobacco.** (1) The hygromycin-resistant shoots of tobacco; (2) the transgenic tobacco in the field; (3) the flower of IbDHKR-overexpressing tobacco **(Left)** and that of wild type **(Right)**; (4) PCR detection on IbDHKR. M: DNA marker; CK: positive control; WT: wild type; the numbers represented the independent transgenic plants; (5) the flowering IbDHKR-overexpressing tobacco plant; (6) the flowering wild type tobacco plant.

In order to investigate the effect of overexpressing *IbDHKR* on anthocyanin biosynthesis, *IbDHKR* was used for genetic transformation of *Nicotiana tubaccum*. Five independent plants were obtained on screening media with hygromycin, but only three of them (Line 1, 7, and 9) had the hygromycin-resistant gene and *IbDHKR* (**Figure 6**), suggesting that they were the authentic transgenic plants. Then the three lines were, respectively, propagated and used for anthocyanin content analysis. The expression of *IbDHKR* was detected in Line 7 and 9, and was not detected in Line 1. Line 9 showed much higher expression level of *IbDHKR* than Line 7. The flowers of Line 7 showed no visible difference with the flowers of wild-type tobacco. This might be due to the low expression level of the transgene *IbDHKR*. Compared with the light pink corolla of wild-type tobacco, the corolla of Line 9 was more highly pigmented (**Figure 6**). In Line 9, the anthocyanin content was about 1.5-fold higher than the wild-type. Overexpression of cranberry DFR in tobacco increased the anthocyanin accumulation in tobacco corolla that kept more highly pigmented than control (Polashock et al., 2002). Because only one transgenic line (Line 9) showed very higher expression level of *IbDHKR*, the definitive conclusion of overexpressing *IbDHKR* on anthocyanin accumulation needed more independent transgenic lines. In fact, overexpression of DFR definitively promoted anthocyanin biosynthesis in many plants (Xie et al., 2004; Lee et al., 2008; Huang et al., 2012; Chu et al., 2014).

## Discussion

Biosynthesis of plant secondary metabolites is spatially and temporally regulated due to specific expression of biosynthetic genes. The storage roots of sweet potato are the active factory to synthesize a lot of secondary metabolites, such as anthocyanins in purple-fleshed sweet potato and carotenoids in orange-fleshed sweet potato. In purple-fleshed sweet potato such as YUZI263, the anthocyanin-biosynthetic genes were highly expressed in storage roots, and with the storage root development, the expression levels of anthocyanin-biosynthetic genes increased. The tissue-preferential/specific patters of biosynthetic genes indicate the involvement of some specific transcriptional control. In transcriptional regulation on anthocyanin biosynthesis, the well-known R2R3 MYB transcription factors (TFs) control the expression of anthocyanin-biosynthetic genes (Li et al., 2016). In purple-fleshed sweet potato, it was the root-specific MYB1 TF (IbMYB1) positively regulating anthocyanin biosynthesis (Mano et al., 2007). Under the positively transcriptional regulation of IbMYB1, the anthocyanin-biosynthetic genes such as *IbF3H. IbDHKR*, and *IbANS* showed very similar tissue profiles and coordinate expression patters. The biosynthetic genes involved in particular pathway are usually coordinately expressed. For example, the artemisinin-biosynthetic genes are specifically expressed in glandular trichomes and positively regulated by methyl jasmonate (Xiang et al., 2015); the tropane-alkaloid-biosynthetic genes are exclusively/preferentially expressed in secondary roots (Xia et al., 2016). In storage roots of purple-fleshed sweet potato, higher expression levels of genes definitively lead to higher levels of anthocyanins. The anthocyanin-biosynthetic genes work together through coordinate expression in storage roots, facilitating anthocyanin biosynthesis.

Metabolic engineering is a promising method to develop new varieties with high-yield target metabolites. For example, overexpression of *SmGGPPS* and/or *SmHMGR* as well as *SmDXS* in transgenic hairy root lines can significantly enhance the production of tanshinone to levels higher than that of the control (Kai et al., 2011). Co-overexpression of *G10H* and *STR* genes caused a 56% increase on the yields of camptothecin compared to non-transgenic hairy root cultures and single gene transgenic lines (Cui et al., 2015). Gene discovery is one of the most important preconditions for engineering pathway. DFR is the key enzyme involved in anthocyanin biosynthesis. The DFR enzymes from different plant species selectively catalyze the reduction of different dihydroflavonol substrates. A*nthurium andraeanum* DFR efficiently reduced DHK, but *Arabidopsis* DFR did not (Leonard et al., 2008). The specific catalysis of dihydroflavonols mediated by DFR was probably due to the amino acid polymorphism especially in the substrate specificity determining region. The substrate specificity determining region was found in IbDHK, composed of T133-K158 (TLNVQPQQKPVYDESCWSDLDFIYAK). The presence of asparagines (N) in this region was assumed to determine the acceptance DHK as substrate (Gosch et al., 2014). In this study, IbDHKR specifically catalyzed DHK reduction, which might be a useful candidate to specifically engineering biosynthesis of pelargonidin-type anthocyanins. The DFR genes are often used to promote anthocyanin biosynthesis. Overexpression of the functionally active DFR enzymes definitively increase anthocyanin accumulation in rice (Takahashi et al., 2006), tobacco (Xie et al., 2004), forsythia (Rosati et al., 2003), crabapple (Tian et al., 2015) and etc.. IbDHKR-overexpressing tobacco also showed thinker pink flowers, suggesting more anthocyanin accumulation in transgenic tobacco plants. In summary, molecular cloning and functional identification of the IbDHKR gene in purple-fleshed sweet potato were helpful to understand anthocyanin biosynthesis in storage roots and provided a candidate gene for engineering anthocyanin biosynthesis.

## Author Contributions

XL, MX, and YF designed and performed most of the experiments. CY provided purple-fleshed sweet potato YUZI263. LZ and QZ managed the plant materials in the field. MC and ZL wrote the manuscript. All authors read and approved the final manuscript.

## Conflict of Interest Statement

The authors declare that the research was conducted in the absence of any commercial or financial relationships that could be construed as a potential conflict of interest.
